# Opportunity Cost in Monetary Donation Decisions to Non-identified and Identified Victims

**DOI:** 10.3389/fpsyg.2019.03035

**Published:** 2020-01-21

**Authors:** Hajdi Moche, Arvid Erlandsson, David Andersson, Daniel Västfjäll

**Affiliations:** ^1^Department of Behavioral Sciences and Learning, Linköping University, Linköping, Sweden; ^2^Department of Management and Economics, Linköping University, Linköping, Sweden; ^3^Decision Research, Eugene, OR, United States

**Keywords:** charitable giving, opportunity cost, donation, decision-making, identified victim, framing

## Abstract

Do people consider alternative uses of money (i.e., opportunity cost) when asked to donate to a charitable cause? To answer this question, we examined the effect of providing versus not providing participants with an opportunity cost reminder when they are asked to donate money to causes with identified and non-identified victims. The results of two studies show that when making one-time donation decisions, people become less willing to donate to charity when reminded of opportunity cost, but mainly for non-identified victims. Moreover, framing the opportunity cost reminder as prosocial versus proself did not influence willingness to donate. Overall, our evidence suggests that opportunity cost reminders influence people’s donation behavior depending on whether charities identify supported victims or not.

## Introduction

Opportunity cost – the benefits a person misses out on when choosing one alternative over another – is a central feature of economic decision making. Classic economic theory suggests that people should consider all options before making purchase decisions (Green, 1894; Palmer and Raftery, 1999). However, decision makers often display opportunity cost neglect – that is, they neglect to take into account alternative ways of spending before a purchase. In a series of experiments, reminding people of opportunity cost was found to make them less willing to buy a consumer item (Frederick et al., 2009). In these studies, opportunity cost became salient simply by adding the text “save the money for other purchases” next to the not-purchase-option. Previous studies investigating opportunity cost suggest that people primarily attend to explicitly presented options (Jones et al., 1998; Greenberg and Spiller, 2016; Plantinga et al., 2018) and thus, opportunity cost neglect occurs when other options (i.e., alternative use of money) remain implicit (Jones et al., 1998; Frederick et al., 2009).

Opportunity cost neglect has up until now mainly been studied for consumer and financial decisions (Jones et al., 1998; Frederick et al., 2009; Greenberg and Spiller, 2016; Plantinga et al., 2018). For example, previous studies on opportunity cost have focused on whether people are willing to buy an item for themselves or not (e.g., Frederick et al., 2009). This refers to personal or proself spending (Dunn et al., 2008), because the purchase predominantly affects (i.e., benefits or harms) only the consumer. However, opportunity cost considerations should also be relevant for other types of financial decisions, such as prosocial spending (i.e., spending that affects other people; Dunn et al., 2008; Aknin et al., 2013; Small and Cryder, 2016). In prosocial spending, opportunity cost can be relevant because people need to trade willingness to spend on other people against other possible uses of money. Although classic economic theory suggests that people seek to maximize their own wealth (i.e., utility-maximization; Becker, 1976), people often put financial self-interest aside to benefit other people in need by, for instance, donating money (Penner et al., 2005; Small and Cryder, 2016). In such situations, opportunity cost reminders may affect the decision to donate money.

Relatedly, willingness to act prosocial (e.g., donate) can decrease when giving is framed as having economic consequences. For example, DeVoe and Pfeffer (2007, 2010) showed that making economic evaluation salient through framing (e.g., charging for time spent working) decreased willingness to donate time (i.e., volunteering) and had a spill-over effect on opportunity cost valuation. Similarly, framing effects have been found and investigated in several domains (e.g., Maule and Villejoubert, 2007) that is of relevance here. In the prosocial domain, framing has affected people’s willingness to act more prosocial in real life settings (Sussman et al., 2015; Sudhir et al., 2016) as well as in economic games (Liberman et al., 2004; Brañas-Garza, 2007; Capraro and Rand, 2018; Tappin and Capraro, 2018). For example, the wording or the adding of a sentence in instructions can affect people’s willingness to act prosocial (Capraro et al., 2019). Liberman et al. (2004) showed that people were more willing to act prosocial when they read that the Prisoner Dilemma was called Community game (i.e., a prosocial frame) than Wall Street game (i.e., a proself frame). Further, Brañas-Garza (2007) showed that adding “the other person relies on you” (i.e., a prosocial frame) in the description of the dictator game made people significantly more generous. Capraro and Vanzo (2019) also found that the wording of the prosocial act (e.g., described as “to not steal” versus “to give”) in the Dictator game affected people’s willingness to give money. Finally, Zlatev and Miller (2016) also showed that willingness to donate differed depending on the frame of the donation appeal and the outcome.

Another type of framing is the content of the task, and to the extent it induces emotional and moral reactions. Compared to proself spending, donation decisions generally involve stronger affective content (i.e., affect-richness, Pachur et al., 2014) as it often involves decisions with life-changing consequences for the recipient of the donation. Thus, framing the content in charity appeals can systematically affect donation decisions (Erlandsson et al., 2018). One example is to include identifiable information in the donation appeal. When a victim is identified (e.g., with a picture and name), people often become more willing to donate than if there is no identified victim, even when holding the cause and need constant (Kogut and Ritov, 2005a, b: Lee and Feeley, 2016; Sudhir et al., 2016). This is called the “identifiable victim effect” (Jenni and Loewenstein, 1997; Kogut and Ritov, 2005a; Genevsky et al., 2013; Västfjäll et al., 2014). The driving mechanism of this effect is the emotional response that identified victims (but not statistical/non-identified victims) evoke in donors, such as distress, sympathy and/or positive arousal (Kogut and Ritov, 2005a; Genevsky et al., 2013; Erlandsson et al., 2015). This empathic response (e.g., Zaki, 2014) makes the donation decision emotionally difficult because it creates a trade-off between the emotional pull evoked by the victim and the opportunities to spend the money differently (Luce et al., 1997; Luce et al., 1999; Rubaltelli and Agnoli, 2017). Similarly, emotional reactions also influence people’s moral judgments (Greene and Haidt, 2002; Bartels, 2008; Wiss et al., 2015). For example, emotional reactions and the perceived moral obligation to donate are often intertwined (Rubaltelli and Agnoli, 2017). However, people sometimes chose not to donate or act prosocial because of a previous moral or prosocial act, a tendency termed moral licensing (Blanken et al., 2015). This licensing can take place even when people have not yet performed the prosocial act, but only considered it (i.e., prospective moral licensing, Cascio and Plant, 2015).

We argue that willingness to spend on charity is a trade-off between benefitting other people and benefitting yourself, in which opportunity cost considerations should be relevant. On the one hand, opportunity cost reminders in a charitable context may, similar to a consumer context, systematically decrease willingness to donate. On the other hand, people may care more about benefitting others that the effect of opportunity cost reminders is small or non-existent in charitable context. The effect of opportunity cost reminders on monetary donations is currently not well understood. The few studies that have studied opportunity cost for prosocial behaviors (DeVoe and Pfeffer, 2010; Knowles and Servátka, 2015; Reed et al., 2016) have mainly investigated other forms of charitable behavior than monetary donations (e.g., the effect of billing time, procrastination and solicitation, or the effect of moral identity) and therefore studied opportunity cost in terms of spending time rather than spending money. Although donating money and time are similar in many ways, there are differences as well. For example, people value money and time differently, and the effort of giving time and money is different (Reed et al., 2016). Thus, it is important to also investigate opportunity cost for monetary donation decisions. This paper will contribute with data on how opportunity cost reminders affect monetary donation decisions to identified and non-identified recipients.

### Hypotheses

Apart from a main effect of opportunity cost reminders (i.e., opportunity cost reminders will overall decrease people’s willingness to donate) and a main effect of identifiability (i.e., identified victims will elicit higher willingness to donate than non-identified victim) our hypotheses are as follows:

H1) We hypothesize that an opportunity cost reminder will decrease people’s willingness to donate when there are no identified victims, but not for identified victims. This interaction effect is the main hypothesis investigated in the two studies in this paper. We base this hypothesis on the fact that identified victims evoke stronger emotional reactions than non-identified victims (Kogut and Ritov, 2005a) and that this emotional reaction will dominate the decision to donate, which in turn can leave little room for people to consider other things, such as how they will spend the money differently. However, since the emotional reaction for affect-poor descriptions of the same need (by using non-identified victims) is less strong, other considerations such as opportunity cost will become relatively more important. Consequently, we predict that the effect of an opportunity cost reminder will be more prominent for non-identified victims than for identified victims. In other words, we believe that people will attend and respond to the opportunity cost reminder more when the emotional reaction is weaker than when it is stronger (i.e., for identified victims).

H2) Secondly, we formulated a hypothesis about the effect of framing the opportunity cost reminder. Previous studies have shown that people change their willingness to donate based on the frame of the appeal, for example by the wording or the adding of a sentence reminding people about their position in relation to another person (e.g., Liberman et al., 2004; Brañas-Garza, 2007; Capraro and Vanzo, 2019). The adding of words is comparable to the opportunity cost reminder that Frederick et al. (2009) included in their experiment. Thus, we predict that the wordings of the opportunity cost reminder can have different effects on willingness to donate (similar to Liberman et al., 2004). More specifically, we predict that people will be less willing to donate to the current charity when framing the opportunity cost reminder as spending on other charities (e.g., “save the money to spend on other charities”) than when framing the reminder as spending on anything else (e.g., “save the money to spend on whatever you want”). Compared to the reminder stating as spending on anything else (i.e., proself frame), people can perceive the reminder stated as spending on other charities (i.e., prosocial frame) as a moral free pass to not donate – similar to prospective moral licensing (Cascio and Plant, 2015). People might feel less morally obligated to donate with a prosocial frame, excusing themselves with thinking “I’m not donating now, but I’ll donate another time” or “I’ll donate to my favorite charity instead”^1^.

We examine the hypotheses both for a single decision (first decision made in a series in Study 1 and as a single-shot decision in Study 2) as well as aggregated across a series of decisions (Study 1). Based on Frederick et al. (2009) the effect of opportunity cost reminder should be larger for the first decision made as several sequentially consumer decisions in itself serve as reminders of alternative uses of money.

Table 1 presents an overview of the hypotheses, test designs, and whether the hypotheses yielded support or not.

**TABLE 1:** Overview of the two studies.

	**Description**	**Test design**	**Hypothesis supported/not supported**
Hypothesis 1	Opportunity cost reminder will decrease willingness to donate mainly for non-identified, but not identified, victims *(And main effects of opportunity cost and identifiability)*	2(Oc-reminder: yes/no) × 2 (Identified victim: yes/no) between-group test design *(Comparing 2 groups: being given or not given an opportunity cost reminder, respective seeing or not seeing an identified victim)*	
**STUDY 1**			
First donation decision	The results of the first decision (out of six), DV = yes/no-decision	2 × 2 contingent table, χ^2^	Supported *(Opportunity cost: Supported Identifiability: Not supported)*
Aggregated donation decisions	The results of the six decisions aggregated, DV = sum of yes-decisions	2 × 2 between-subject, ANOVA	Not supported *(Opportunity cost: Supported*, *Identifiability: Not supported)*
**STUDY 2**			
Donation decision	The results of a one-time donation decision, DV = yes/no-decision	2 × 2 contingent table, χ^2^	Supported *(Opportunity cost: Not supported, Identifiability: Supported)*
Donation amount	The results for donation amount, DV = amount donated	2 × 2 between-subject, ANOVA	Supported for analyses including all participants/Not supported for analyses including only those that donated
Hypothesis 2	A prosocial framed opportunity cost reminder will decrease willingness to donate more than a proself framed reminder	Prosocial opportunity cost reminder is compared with proself opportunity cost reminder	
**STUDY 1**			
First donation decision	The results of the first decision (out of six), DV = yes/no-decision	2 frames (prosocial versus proself) are compared, χ^2^	Not supported
Aggregated donation decisions	The results of the six decisions aggregated, DV = sum of yes-decisions	2 frames (prosocial versus proself) are compared, χ^2^	Not supported
**STUDY 2**			
Donation decision	The results of a one-time donation decision, DV = yes/no-decision	2 frames (prosocial versus proself) are compared, χ^2^	Not supported
Donation amount	The results for donation amount, DV = amount donated	2 frames (prosocial versus proself) are compared, between-subject ANOVA	Not supported

**DV = Dependent variable.**

## Study 1

### Materials and Methods

The hypotheses, experimental design and sample size has been pre-registered and can be found at https://osf.io/2tdeu/

#### Participants

1,277 Swedish adults were recruited online through Origo Group, an independent research company with a roughly representative panel of Swedish adults. However, the data of 134 participants was excluded because they had stated an age that was outside our intended age range (18–70 years)^2^. Thus, our final sample constituted of 1,143 participants (50.7% women, *M*_age_ = 45.8, *SD* = 14.5).

#### Design

The study has a 2 (identifiability) × 3 (opportunity cost frame) between-group experimental design. The key dependent variable is willingness to donate. Participants were randomized to one of six conditions.

##### Identifiability

Half of the participants read descriptions of charity causes with an identified victim included. Identification was done with a name, age and a photo of a victim (see Appendix)^3^. The other half of the participants, who were randomized to the non-identified conditions, only read information about the charity cause (based on Small et al., 2007 and Study 3 in Erlandsson et al., 2016).

##### Opportunity cost

In four of the six conditions, an opportunity cost reminder was included when participants would indicate their choice. Participants would next to the option “No” either read “Save the money to spend on whatever you want” (proself frame) or “Save the money to spend on other charity causes” (prosocial frame). The remaining conditions had no opportunity cost reminder: the options were only “Yes” or “No.”

#### Procedure

After giving consent, participants read a brief description of the study, informing them that they would see six scenarios and be asked to donate to these presented charitable cause. Every participant then read six different scenarios (in randomized order) containing information about a specified charity cause. The six causes were breast cancer, clean water, trafficking, war refugees, diabetes, and bullying (no specified charity organization). We chose the six causes mainly based on trustworthiness (common charity causes). After reading each scenario, participants indicated if they were willing to donate a specified amount (ranging from 75 to 125 SEK) to the cause (yes/no), before moving on to read next scenario. The reason for the fixed amount and the dichotomous decision was to keep it as the opportunity cost manipulation by Frederick et al. (2009). After reading and responding to the six scenarios, participants answered four additional rating measures, concerning their experiences of and judgments about each scenario (not reported here but can be found in Appendix). At the end of the survey, participants responded to demographic questions about their gender, age, level of education, political orientation, number of adults and children in their household and their household’s monthly income. Participants also responded to a single item question about purchasing a cell phone (same as Frederick et al., 2009), which they saw after the scenarios (not reported here but can be found in Appendix).

#### Data Analysis

Participants responded to a total of six sequentially presented decisions, and as it is likely that the first decision made may differ from the aggregated decisions across all six conditions, we will present the results separately. The results of the first decision made demonstrate participant’s responses as if they only saw and responded to one scenario (similar to the method used in most studies in opportunity cost and charitable giving, e.g., Kogut and Ritov, 2005a; Frederick et al., 2009). Here, we will analyze willingness to donate by chi-square tests (percentages of participants responding yes to the donation appeal). When presenting the results on aggregated level, our dependent variable is the total sum of responses, representing participants’ aggregated willingness to donate (ranging from 0 to 6 for each participant) using ANOVAs.

### Results

We present the results in two parts. First, the results for the first decision made will be presented. These results will only be presented on condition level, because of the small number of observations for each single scenario. Second, the results for the aggregated decisions will be presented, both on condition levels and for single scenarios. The result sections will primarily focus on our main hypothesis.

However, first we present the results of our second hypothesis, where we predicted that willingness to donate would be less when the reminder was framed to spend on other charities (i.e., prosocial frame) than to anything else (i.e., proself frame). This hypothesis yielded no support, neither for the first decision, χ^2^(1) = 0.01, *p* = 0.91, main effect on aggregated level, χ^2^(1) = 0.01, *p* = 0.92, or on scenario level: breast cancer (*p* = 0.45), clean water (*p* = 0.38), trafficking (*p* = 0.69), war refugee (*p* = 0.35), diabetes (*p* = 0.42) and bullying (*p* = 0.53). Hence, no support for H2 was found and the two opportunity cost frames were combined into a single, general opportunity cost reminder factor. This leads us to have a 2 × 2 between-group study design when we investigate the interaction effect.

#### Results for the First Decision

Table 2 shows an overview of the results according to the first decision made by participants. The percentages of participants willing to donate categorized by conditions (columns) and scenarios (rows) are presented.

**TABLE 2:** Percentage of participants willing to donate in the first decision, for conditions and scenarios.

	**Non-identified**			**Identified**		
**Helping**	**Oc:**	**Oc:**	**No-oc**	**Oc:**	**Oc:**	**No-oc**
**situation**	**prosocial**	**proself**	***N* = 192**	**prosocial**	**proself**	**Af = 184**
	***N* = 196**	***N* = 188**		**Af = 190**	**Af = 193**	
Breast cancer	47.4%	50%	65.5%	45.9%	48.6%	56.5%
Clean water	54.5%	41.5%	65.2%	57.7%	60.9%	75.8%
Trafficking	52.8%	48.0%	83.3%	48.5%	48.4%	51.7%
Refugee	63.0%	40.0%	60.7%	35.3%	33.3%	44.4%
Diabetes	37.5%	60.0%	60.5%	25.0%	44.7%	48.4%
Bullying	46.7%	48.3%	57.6%	60.7%	43.2%	59.4%
Total	50.0%	47.9%	65.6%	44.7%	46.1%	56.0%

**Oc:prosocial, Opportunity cost reminder, stated as “Save the money to spend on other charity causes”; Oc:proself, Opportunity cost reminder, stated as “Save the money to spend on whatever you want”; No-oc, No opportunity cost reminder.**

**FIGURE 1:**
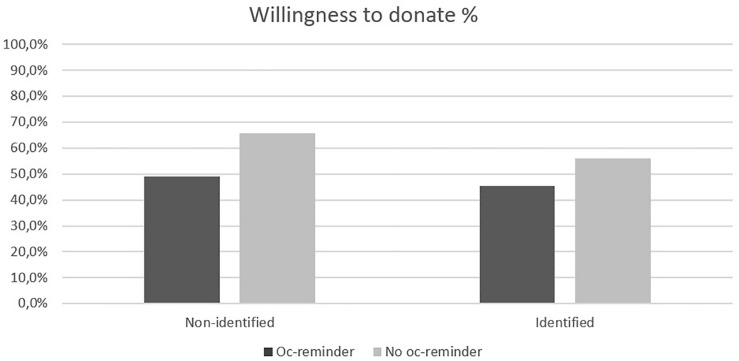
Percentages of willingness to donate for the two factors identifiability (Non-identified or Identified) and opportunity cost (Oc-reminder or No oc-reminder) for the first decision.

As expected, there is a negative main effect of opportunity cost reminder, χ^2^(1) = 19.0, *p* < 0.001. 60.9% of participants who did not receive a reminder were willing to donate whereas only 47.2% of participants who received a reminder were willing to donate (i.e., a 13.7% difference). Also, interestingly, we found no significant effect of victim identifiability, χ^2^(1) = 3.67, *p* = 0.056. People seeing identified victims were less willing to donate (48.9%) than people who did not see identified victims (54.5%), meaning the results went in the opposite direction from our prediction. However, more important, we found an interaction effect between opportunity cost and identifiability, χ^2^(3) = 23.4, *p* < 0.001. This can be seen in Figure 1. The difference between receiving or not receiving an opportunity cost reminder was larger for non-identified victims [Oc-reminder: 49.0% versus No oc-reminder: 65.6%, χ^2^(1) = 14.3, *p* < 0.001] than for identified victims [Oc-reminder: 45.4% versus No Oc-reminder: 56.0%, χ^2^(1) = 5.53, *p* = 0.019]. This result supports H1.

#### Results for the Aggregated Decisions

Table 3 shows an overview of the results for the responses to all six decisions aggregated, presenting the percentages of participants willing to donate categorized by conditions (columns) and scenarios (rows).

**TABLE 3:** Percentage of participants willing to donate over all decisions, for conditions and scenarios.

	**Non-identified**			**Identified**		
**Helping situation**	**Oc: prosocial *N* = 196**	**Oc: proself *N* = 188**	**No-oc *N* = 192**	**Oc: prosocial *N* = 190**	**Oc: proself *N* = 193**	**No-oc *N* = 184**
Breast cancer	56.5%	56.3%	57.0%	53.1%	50.7%	57.1%
Water	59.3%	58.2%	65.0%	60.2%	67.6%	57.1%
Trafficking	48.6%	56.8%	62.1%	52.1%	54.5%	52.9%
Refugee	45.8%	41.3%	51.4%	44.1%	45.1%	43.8%
Diabetes	39.4%	40.8%	39.7%	29.4%	34.7%	39.5%
Bullying	45.4%	44.6%	49.1%	53.1%	50.2%	57.1%
Total	49.2%	49.7%	54.0%	48.7%	50.5%	51.3%

**Oc:prosocial, opportunity cost reminder, stated as “Save the money to spend on other charity causes”; Oc:proself, opportunity cost reminder, stated as “Save the money to spend on whatever you want”; No-oc, no opportunity cost reminder.**

**FIGURE 2:**
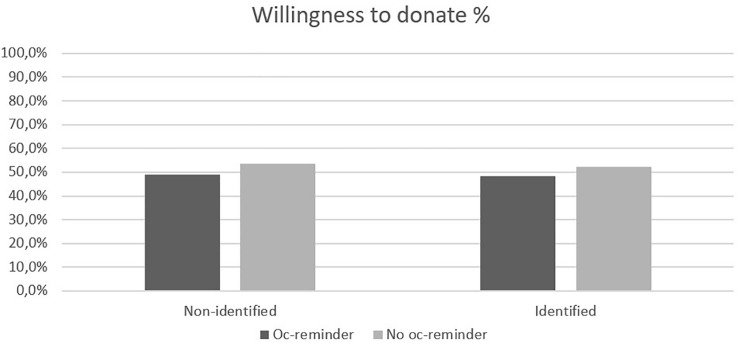
Percentages of willingness to donate for the two factors identifiability (Non-identified or Identified victims) and opportunity cost (Oc-reminder or No oc-reminder) for all six donation decisions aggregated.

Unlike the first decision, we did not find a significant interaction between opportunity cost reminder and victim identifiability at the aggregated level (see Figure 2), χ^2^(3) = 4.00, *p* = 0.26. This was also true on scenario-level after Bonferroni correction. Further, as expected, there was a significant main effect of opportunity cost reminder at the aggregated level [mean of donation-responses, No oc-reminder: *M* = 3.18, *SD* = 2.03 versus Oc-reminder: *M* = 2.92, *SD* = 1.96, *F*(1, 1141) = 4.44, *p* = 0.035, η*_*p*_*^2^ = 0.004]. This indicates that participants become significantly less willing to donate when they were reminded of opportunity cost (48.7%), compared to when they were not reminded (53.1%). However, the difference is 4.4%, suggesting a rather small effect size (compared to 13.7 percent for the first decision). Specifically, the decrease in willingness to donate from one decision to aggregated decisions is mainly found in the control condition (60.9% donated for one decision versus 53.1% for aggregated decisions in control conditions). On scenario-level, no significant results emerged after Bonferroni correction. Further, the main effect for victim identifiability on aggregated level was not significant [Identified: *M* = 2.98, *SD* = 2.04 versus Non-identified: *M* = 3.03, *SD* = 1.93, *F*(1, 1141) = 0.20, *p* = 0.66].

When investigating the results for the identifiable victim hypothesis for the six separate scenarios, we noticed that only two causes (clean water and bullying) went in the predicted direction (higher willingness when an identified victim was present). Interestingly, these were the only scenarios depicting a child as the identified victim. Thus, as a *post hoc* analysis, we tested for an interaction effect for willingness to donate (%) between identifiability and victim category (child versus adult). The result of this *post hoc* interaction analysis yielded a significant result both for the first decision, χ^2^(3) = 16.1, *p* = 0.001, and all decisions aggregated, χ^2^(3) = 47.9, *p* < 0.001. This indicated that identifying information increased willingness to donate for child victims but not adult victims. We also asked another sample (*N* = 71, 52.1% women, *M_age_* = 27.1) to rate the pictures, in a within-subject design with different orders, on perceived attractiveness, needy-looking and sympathy-evoking on a Likert scale from 1 (= *Very little)* to 10 (= *Very much).* The result of a repeated-measure ANOVA showed that the pictures of the children, in comparison with adults, were perceived as more attractive, *F*(1, 70) = 8.42, *p* = 0.005, η*_*p*_*^2^ = 0.11, more needy, *F*(1, 70) = 85.6, *p* < 0.001, η*_*p*_*^2^ = 0.55, and more sympathy-evoking, *F*(1, 70) = 64.5, *p* < 0.001, η*_*p*_*^2^ = 0.48.

### Discussion Study 1

This study set out to examine how opportunity cost reminders affect donations to identified and non-identified victims. We predicted that opportunity cost reminders would decrease donations, and we did indeed find support for this, especially when investigating the first decision made. Further, we predicted, but did not find, that identified victims would yield higher donations than non-identified victims. Most importantly, we found an interaction effect between opportunity cost reminder and victim identifiability for the first decision made (but not for all decisions aggregated). Also, we predicted, but did not find evidence for a framing effect of the opportunity cost reminder.

This study is, to our knowledge, the first to investigate opportunity cost for monetary donation decisions. The results show that reminding people of opportunity cost, prior to their donation decision, decreases their willingness to donate. The results for the first decision are in line with the findings from proself spending (Frederick et al., 2009; Plantinga et al., 2018), and also how people react to economic evaluations when asked to donate time (DeVoe and Pfeffer, 2010). However, the opportunity cost effect decreased from 13.7 to 4.4% when participants made sequential donation decisions. Interestingly, the biggest decrease in willingness to donate was found in conditions without an opportunity cost reminder. One explanation for this decrease is that the study’s design implicitly evoked opportunity cost considerations; participants might have been reminded of other donation opportunities when seeing several donation appeals. This is consistent with Study 4 in Frederick et al. (2009), showing that listing other ways of spending money prior to a purchase served as an implicit opportunity cost reminder. When showing and asking people to make decisions about several appeals, they may either become less willing to help or post-pone making a decision at all (Knowles and Servátka, 2015). Another possibility is that making multiple donation decisions leads to emotional fatigue (Slovic, 2007) or cognitive overload (Tinghög et al., 2016), which then leads to lessened willingness to donate. This is also consistent with research showing that people’s willingness to help decreases when they see other victims or causes that cannot be helped, an effect called pseudo-inefficacy (Västfjäll et al., 2015). This can have important implications for charity organizations if overall willingness to donate decreases when potential donors see different causes or means of helping.

Contrary to many studies that finds a positive effect of identifiability on donations (Jenni and Loewenstein, 1997; Kogut and Ritov, 2005a, b; Genevsky et al., 2013), we did not find such an effect. The failure to find the identifiable victim effect is not unique to this study (Wiss et al., 2015; Hart et al., 2018). Further, the identifiable victim effect might be explained by factors included in the appeal, such as the content and description of the victim(s). For example, we found this effect for scenarios depicting children, but not for adult victims. Further, we found that children (compared to adults) elicited higher ratings of attractiveness, neediness and evoked sympathy, which is in line with research showing that children and perceived innocence of the victims are predictors of the identifiable victim effect (Kogut, 2011; Lee and Feeley, 2016; James and Zagefka, 2017). However, future research should investigate the child-adult difference in the identifiable victim effect by keeping factors such as the donation scenario constant.

Importantly though, in line with our main hypothesis, we found that the effect of opportunity cost reminders was larger for non-identified than for identified victims for the first decision made (but not for all decisions aggregated). Thus, we find some support for the notion that the affective pull of an identified victim may sometimes counter the negative effect of opportunity cost reminders. This study thus gives a first indication that the effect of opportunity cost reminder on one-time donation decisions is most evident for charity appeals that do not elicit strong emotional reactions.

## Study 2

Study 2 extends our initial findings from Study 1 in two ways. First, we allow participants to specify the amount they would like to donate rather than ask them whether they would like to donate a certain amount. Second, we employ two revised opportunity cost frames that are more clearly separated. The opportunity cost reminder used in Study 1 (i.e., “save money to spend on anything else”) implied the possibility of donating to an alternative charity, leading to an ambiguous framing that could be interpreted as prosocial or proself. This study was pre-registered and can be found at https://osf.io/2tdeu/.

### Materials and Methods

#### Participants

1,254 Swedish adults were recruited online through Origo Group, an independent research company with a roughly representative panel of Swedish adults (51.8% women, *M*_age_ = 42.4, *SD* = 14.4).

#### Design

The study used a 2 (identifiability) × 3 (opportunity cost frame) between-group experimental design. Participants were randomized to one of six conditions. There were two dependent variables: willingness to donate and donation amount.

##### Identifiability

The description of the charity cause either included a description of an identified victim or not, similar to Study 1. Half of the participants read a description where an identified victim was included, and the other half only read information about the charity cause. Identification was done with a name, age and a photo of a victim (see Appendix).

##### Opportunity cost

Two versions of an opportunity cost reminder were included. Participants in the prosocial frame condition were presented with the following two choices in their donation decision: “Yes, I would like to donate” and “No, I will save the money to spend on *other charitable causes.*” Participants in the proself frame condition were presented with “Yes, I would like to donate” and “No, I will save the money to spend on *my future purchases.*”

#### Procedure

After reading a short description of the study and giving consent, participants were asked to imagine that they were at a grocery store check-out and being asked if they are willing to donate to a charity organization that works to minimize bullying. The description of the bullying scenario was the same as in Study 1 (both identified and non-identified), but with some small adjustments in wording to better suit the scenario they were asked to imagine (see Appendix). The reason we chose this scenario among the six scenarios from Study 1 was mainly because approximately 50% of participants were willing to donate to it, both with and without opportunity cost reminder as well as for identified or non-identified victim, allowing for a fair test of the experimental manipulations. Also, this was one of the scenarios from Study 1 where the identifiable victim effect was evident, allowing us the greatest chance to find the hypothesized interaction effect with opportunity cost reminder.

Participants who indicated they were willing to donate in the scenario were directed to a page where they were asked how much they would like to donate [“*State how much you would like to donate. (max 500 SEK)*”]. Participants that chose not to donate were not asked to indicate how much they would like to donate. At the end of the survey, participants responded to demographic questions about gender and age before they were debriefed and thanked for their participation.

### Results

Similar to Study 1, we found no significant results for the framing hypothesis, suggesting that the two frames of the opportunity cost reminder would yield different results. People were similarly willing to donate when being reminded with a prosocial frame (56.7%) and with a proself frame (57.7%), χ^2^(1) = 0.08, *p* = 0.78. Also, there was no significant difference in donation amount between the prosocial frame (*M* = 52.1, *SD* = 81.2) and proself frame (*M* = 57.9, *SD* = 94.6), *F*(1, 834) = 0.88, *p* = 0.35. Hence, no support for H2 was found and the two opportunity cost frames were combined into a single, general opportunity cost reminder factor. Thus, we have a 2 × 2 between-group study design when we investigate the interaction effect between opportunity cost reminder and identifiability.

Table 4 shows the percentages and donated amount for the different conditions for this study. Donated amount includes only participants that indicated they were willing to donate (i.e., donors).

**TABLE 4:** Percentages choosing to donate and donated amount for the conditions.

	**Non-identified**		**Identified**		***Total***	
	**%**	**SEK (*SD*)**	**%**	**SEK (*SD*)**	**%**	***SEK* (*SD*)**
No oc-reminder	60.6%	88.2 (97.6)	62.0%	98.8 (114.6)	61.2%	93.5 (106.3)
Prosocial oc-reminder	50.5%	86.3 (75.4)	63.0%	96.5 (99.2)	56.7%	91.9 (89.3)
Proself oc-reminder	49.0%	98.0 (111.1)	66.3%	102.1 (102.5)	57.7%	100.3 (106.1)
Total	53.4%	90.6 (95.7)	63.8%	99.2 (105.2)		

**No oc-reminder, no opportunity cost reminder; Prosocial oc-reminder, Opportunity cost reminder, stated as “Save the money to spend on other charitable causes”; Proself oc-reminder, Opportunity cost reminder, stated as “Save the money to spend on my future purchases.”**

**FIGURE 3:**
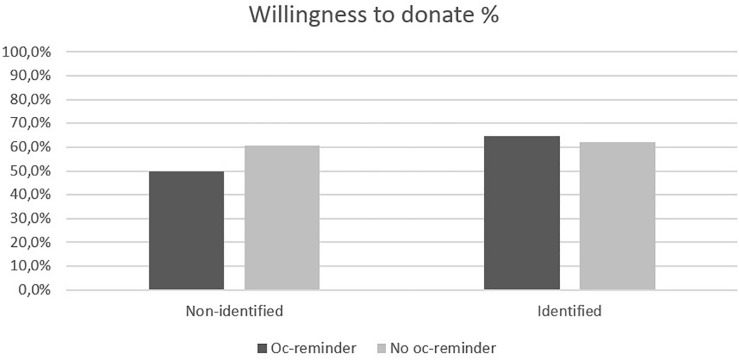
Percentages of willingness to donate for the two factors identifiability (Non-identified or Identified) and opportunity cost (Oc-reminder or No oc-reminder) for the single scenario.

Contrary to our prediction, there was no significant effect of opportunity cost reminder for willingness to donate, χ^2^(1) = 1.90, *p* = 0.17. People receiving an opportunity cost reminder (57.2%) were not significantly less willing to donate than people not receiving a reminder (61.2%). Further, in line with our prediction, we found an identifiable victim effect, χ^2^(1) = 13.9, *p* < 0.001. People seeing an identified victim were significantly more willing to donate (63.8%) than people who did not see an identified victim (53.4%). Most importantly, we found a significant interaction effect between opportunity cost reminder and identifiability, χ^2^(3) = 21.1, *p* < 0.001 (see Figure 3). While the opportunity cost reminder reduced willingness to donate for non-identified victims [Oc-reminder = 49.8% versus No oc-reminder = 60.6%, χ^2^(1) = 6.63, *p* = 0.010], there was no significant effect in the case of identified victim [Oc-reminder = 62.0% versus No oc-reminder = 64.7%, χ^2^(1) = 0.44, *p* = 0.51]. This supports H1.

**FIGURE 4:**
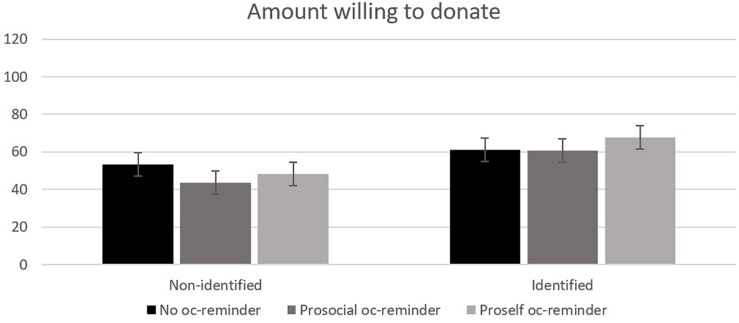
Mean donation amount (SEK) among all participants, both donors and non-donors, divided on the two factors identifiability (Non-identified or Identified) and Opportunity cost (No oc-reminder, Prosocial oc-reminder or Proself oc-reminder). Error bars represent standard error of the mean.

We obtained consistent results using the donation amount as an alternative dependent variable^4^. Figure 4 shows the mean amount donated when all participants were included.

Again, a significant interaction effect was found, *F*(3, 1250) = 3.24, *p* = 0.022, η*_*p*_*^2^ = 0.008. Participants that were reminded of opportunity cost and saw a non-identified victim donated significantly less (*M* = 45.8, *SD* = 81.1) than participants that were reminded and saw an identified victim (*M* = 64.3, *SD* = 93.9).

#### Discussion Study 2

This study extended and replicated Study 1 by investigating the effect of two opportunity cost reminders with or without identified victims for a one-time donation decision, both for a yes/no decision to donate and for donation amount. We found no general opportunity cost effect for willingness to donate, but we found a significant identifiable victim effect. Importantly, we found a significant interaction effect between opportunity cost and identifiability – the effect of opportunity cost reminder was stronger for non-identified victims than for identified victims. Further, we found no difference in willingness to donate if the opportunity cost reminder was framed to prosocial causes (“other charitable causes”) or to proself causes (“my future purchases”). Finally, we obtained consistent results with the second dependent variable, donation amount.

In contrast to results for proself spending domain (Frederick et al., 2009), we found no overall difference in willingness to donate between receiving an opportunity cost reminder and not receiving it for the specific scenario used here. Further, we found a main effect of identifiability. Using a version of the bullying scenario from Study 1, we replicate that the identified victim received a higher level of willingness to donate than the non-identified victim. This suggests that the identifiable victim effect is rather robust for this specific scenario.

More importantly though, we found an interaction effect. This was in line with our prediction, stating that an opportunity cost reminder will decrease willingness to donate mainly for appeals with little affective content. This suggest that an opportunity cost reminder affect people mainly when the emotional pull from the purchase item (i.e., the charity cause) is weaker. This interaction effect could also explain the results from Frederick et al. (2009) as it is likely that the purchase items used in their studies (e.g., a DVD) induce relatively weak emotions, especially compared to an identified victim.

The results of this study also show that the framing of the opportunity cost reminder does not seem to influence willingness to donate as we had predicted, even though we formulated the frames more stringently to eliminate the possible confounding these two frames had in Study 1. Opportunity cost reminders that highlight opportunities to spend on other charities or on own future purchases did not affect people’s willingness to donate differently. While this was not what we had predicted, this finding is in line with studies that failed to replicate that adding or changing the wording affects willingness to act prosocial (Ellingsen et al., 2012; Dreber et al., 2013; both trying to replicate Liberman et al., 2004).

## General Discussion and Conclusion

We have conducted two studies that investigated the effect of opportunity cost reminders on willingness to donate to identified and non-identified victims. The main result of the studies is an interaction effect between opportunity cost reminder and identifiability of the victim. The results show that for a one-time donation decision, people become less willing to donate if they are reminded of opportunity cost and see a non-identified, rather than an identified victim. However, our first study shows that when people make the decision repeated times, this effect is diminished. The studies also show mixed results for the main effects of identifiability and opportunity cost reminder. Finally, both studies found no effect of whether the opportunity cost reminder is framed as prosocial or proself.

In both studies, we find an interaction effect for one-time donation decisions. People seeing a non-identified victim were more affected by an opportunity cost reminder than people seeing an identified victim. People’s willingness to purchase an item for themselves, proself spending, decreases when they are reminded of other ways to spend money (Frederick et al., 2009), and to some extent we found similar results in the domain of prosocial spending. We found that an opportunity cost reminder decreased people’s willingness to donate in both studies (both for one-time and repeated decisions), but the main effect of opportunity cost was not significant in the second study. Instead, we found an effect of opportunity cost reminder mainly for non-identified victims. Thus, one possible conclusion is that opportunity cost reminders might not generally decrease people’s willingness to spend money in prosocial decisions, it may instead depend on the affect-richness of the decision (Luce et al., 1997, 1999; Pachur et al., 2014).

The results for the identifiable victim manipulation in these two studies were mixed. In the first study, there was a trend toward a reversed identifiable victim effect for the first decision (but not for repeated decisions), whereas in the second study, the identifiable victim effect was evident. However, the scenario used in the second study was deliberately chosen to increase chances of finding this effect. A conclusion is that the identifiable victim effect is dependent on the scenario used in the donation appeals. According to the results for the ratings of the victim pictures from the additional sample in Study 1, this seems to depend on the perceived neediness, attractiveness and evoked sympathy of the victim. These attributes also covaried with whether the victim was a child or an adult. Although a previous meta-analysis showed that children victims are more likely to elicit the identifiable victim effect (Lee and Feeley, 2016) and many studies investigating this effect has depicted the victim as a child (e.g., Kogut and Ritov, 2005a; Small et al., 2007; Erlandsson et al., 2015), this interaction needs more rigorous investigation before it is possible to conclude that the identifiable victim effect is stronger for child than adult victims.

Across both studies, we found no systematic effect of framing the opportunity cost reminder as prosocial or proself. Although many previous studies have found effects for similar type of frames (e.g., Petrinovich and O’Neill, 1996; Liberman et al., 2004; Sussman et al., 2015; Capraro and Vanzo, 2019), there are studies that have not (e.g., Ellingsen et al., 2012; Dreber et al., 2013). For example, Tappin and Capraro (2018) found a general moral framing effect but no effect of whether the moral frame was highlighting “doing good” or “avoid bad.” Also, Palmiotti et al. (2019) found that framing had no effect on moral choices, but did affect subjective unpleasantness from making the decision. Even though prosocial versus proself spending have very different emotional consequences (Dunn et al., 2008), our results suggest that people’s donation decisions are not affected by whether they are reminded of prosocial or proself opportunity costs.

We conclude that being reminded of alternative uses of money decreases willingness to donate to non-identified victims more than to identified victims. Our results also suggest that the identifiable victim effect is contingent on the scenario used and, more specifically, the attributes of the victim such as whether the victim is a child or adult, where the former appear to elicit stronger sympathy and is perceived as more needy. Last, we find no systematic framing effect of whether the opportunity cost reminder was prosocial or proself.

### Limitations

Although this study presents some novel results on the effect of opportunity cost reminders in a prosocial domain, there are some limitations. The paradigm used to investigate opportunity cost neglect in the present research was adopted from Frederick et al. (2009), but it is important to note that this is just one way of making opportunity cost salient. Thus, it is possible to use other manipulations to remind people of opportunity cost than what have been done in this study. Further, our studies (similar to Frederick et al., 2009) used hypothetical decision, so an important task for future research is to examine if the current findings will hold up for real donations.

## Data Availability Statement

The datasets generated for this study are available on request to the corresponding author.

## Ethics Statement

Ethical review and approval was not required for the study on human participants in accordance with the local legislation and institutional requirements. The patients/participants provided their written informed consent to participate in this study.

## Author Contributions

HM designed the experiments, collected and analyzed the data, and wrote the manuscript. AE contributed to the manuscript revisions. DA analyzed some of the data. DV initiated the project, designed the experiments, and wrote and revised the manuscript.

## Conflict of Interest

The authors declare that the research was conducted in the absence of any commercial or financial relationships that could be construed as a potential conflict of interest.

## Footnotes

1 Related to H2, we also tested the possibility of an interaction effect between opportunity cost framing and identifiability. These results can be found in the Appendix (found at https://osf.io/2tdeu/).

2 To test if the excluded participants differed from the rest of the sample in some aspect, an exclusion analysis was conducted. These tests revealed no significant results. For example, no significant difference for gender was found. Number of excluded women (10.6%) and men (10.1%) did not differ from each other, nor did the ratio of women between the excluded and the total sample significantly differ (*p* = 0.73). Similarly, no significant differences were found for political orientation, education level or income.

3 https://osf.io/2tdeu/

4 The presented results of this dependent variable include both donors and non-donors (participants who indicated they were not willing to donate). We coded non-donors as donating 0 SEK. Although not reported in the pre-registration, we thought this way was important since it includes all participants in the analyses. The main effect of identifiability with all participants included was significant, *F*(1, 1252) = 8.55, *p* = 0.004, η*_*p*_*^2^ = 0.007. Participants seeing an identified victim donated significantly more (*M* = 63.3, *SD* = 96.6) than participants not seeing an identified victim (*M* = 48.4, *SD* = 83.2). Further, the main effect of opportunity cost reminder with all participants included was non-significant, *F*(1, 1252) = 0.17, *p* = 0.68. However, we also ran the analyses with donors only and for these analyses, the interaction effect was not evident, *F*(3, 730) = 0.48, *p* = 0.70, as well as main effect of opportunity cost reminder, *F*(1, 732) = 0.12, *p* = 0.73, and the main effect of identifiability, *F*(1, 732) = 1.33, *p* = 0.25.
