# Supplementary material for: Opportunity Cost in Monetary Donation Decisions to Non-identified and Identified Victims
Source: Front Psychol. 2020 Jan 21;10:3035. doi: 10.3389/fpsyg.2019.03035 (PMC6986473; doi:10.3389/fpsyg.2019.03035)
Supplement: Supplementary file 1 [file Table_1.DOCX]

**Appendix –
Opportunity cost in monetary donation decisions to non-identified and identified victims**

**Study 1**

**Scenarios (translated)**

***Unidentified scenarios [other charities frame]***

*Scenario 1 – Breast cancer non-identified*

You will in this scenario decide if you want to donate to an organization that works to fight cancer.

You read about a charity organization that works to help people with cancer and to prevent cancer. In the ad you read that during this year the charity organization will work directed to breast cancer. They will help cancer patients and their families to get access to treatment rapidly. In the charity organization’s directed work, they will also contribute to research that aims to find more effective treatments and to prevent the emergence of breast cancer. In the ad it says that private persons can donate to this purpose. By donating 125 SEK, you will contribute to people with cancer to receive fast treatment and to research about breast cancer.

Do you choose to donate to the purpose breast cancer treatment?

- Yes
- No [save money to use for other charitable purposes]

*Scenario 2 – Clean water non-identified*

You will in this scenario decide if you want to donate to an organization that works to increase accessibility to clean water at places where this is missing.

Access to clean water is something that is missing in a lot of places in the world. This has resulted in and will continue to result in deaths that could have been prevented with clean water. You are reading about an organization that works to build water sources and treatment plants where these are still missing. They have a campaign where you as a private person can donate to their ongoing project. By donating 115 SEK you contribute to the building of one treatment plant in one region.

Do you choose to donate to the purpose clean water?

- Yes
- No [save money to use for other charitable purposes]

*Scenario 3 – Trafficking non-identified*

You will in this scenario decide if you want to donate to an organization that works to minimize trafficking and help victims of trafficking.

You are reading about a charity organization that works to minimize cases of trafficking and help victims of trafficking to escape their situation. This charity organization educates people to identify situations of trafficking and equips field workers to help trafficking victims to escape their situation. By donating 75 SEK you contribute to educating field workers that actively will work to help victims of trafficking.

Do you choose to donate to the purpose trafficking?

- Yes
- No [save money to use for other charitable purposes]

*Scenario 4 – Refugee victim non-identified*You will in this scenario decide if you want to donate to an organization that helps people who has escaped war.

You are reading about a charity organization that works to help children, adults and families who have fled from their home country because of war. This charity organization aids refugees with shelter, necessities and safety in different refugee camps. By donating to this organization more people on the run can survive when they no longer have a permanent accommodation, income or access to necessities. You are reading about the possibility to donate to this purpose as a private person. By donating 120 SEK you contribute that more refugees can be helped with accommodation and necessities.

Do you choose to donate to the purpose war refugees?

- Yes
- No [save money to use for other charitable purposes]

*Scenario 5 – Diabetes non-identified*

You will in this scenario decide if you want to donate to an organization that helps children and adults with diabetes.

Many children and adults with diabetes are affected negatively in many ways in their everyday life. An organization that works for people with diabetes helps them to get support in their everyday life, for example by facilitate health care visits, create networks and inform about how to ease their everyday life, alleviate their symptoms and prevent serious injuries. This charity organization also contributes to research about diabetes. You are reading that you as a private person can contribute to their work. By donating 85 SEK you contribute to helping children and adults with diabetes to get support, help and information as well as to research about diabetes.

Do you choose to donate to the purpose diabetes?

- Yes
- No [save money to use for other charitable purposes]

*Scenario 6 – Bullying non-identified*

You will in this scenario decide if you want to donate to an organization that works to reduce bullying at schools.

You are reading about an organization that in different ways works with anti-bullying at schools around the country. This charity organization works with educating and implementing work methods that reduces bullying among children. They are also working to eliminate bullying in specific cases at schools. By donating 100 SEK you contribute to the work with reducing cases of bullying at schools and help victims of bullying to have a better school situation.

Do you choose to donate to the purpose anti-bullying?

- Yes
- No [save money to use for other charitable purposes]

***Identified scenarios [whatever else framing]***

*Scenario 1 – Breast cancer, identified*

You will in this scenario decide if you want to donate to an organization that works for fighting cancer.

You are currently reading about Marielle, 42 years old, who have been diagnosed with breast cancer since 4 months back. It has been a hard time for her and her family. Marielle is still waiting to get treatment for her breast cancer.

You have the possibility to donate to a charity organization that works for the cause breast cancer. The charity organization is now working directed to breast cancer; to help patients with cancer and their families to get access to fast treatment as well as contribute to research that aims to find more effective treatments and to prevent the occurrence of breast cancer. By donating 125 SEK you contribute for people with cancer, like Marielle, to get access to fast treatment as well as to research about breast cancer.


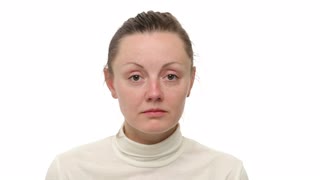


*Marielle, 42 years*

Do you choose to donate to the purpose breast cancer treatment and help victims like Marielle?

- Yes
- No [save money to use to whatever you want]

*Scenario 2 – Clean water, identified*You will in this scenario decide if you want to donate to an organization that works to increase access to clean water at places where this is missing.

You have just heard about 7-year old Micaels life story. He lives in an area where he and his family don’t have access to clean water. Instead they need to walk several miles to possibly get water from a water well, that they then must spare with. Micael has now started walking by himself to get clean water. Many times, he has needed to stop along the way because of exhaustion.

Access to clean water is something that is missing in many places in the world. It has resulted in and continues to result in deaths, that could have been prevented with clean water. You have the possibility to donate to a charity organization that works with building water sources and treatment plants where these are still missing. By donating 115 SEK you contribute to the building of one treatment plants in one region, that can help persons like Micael.


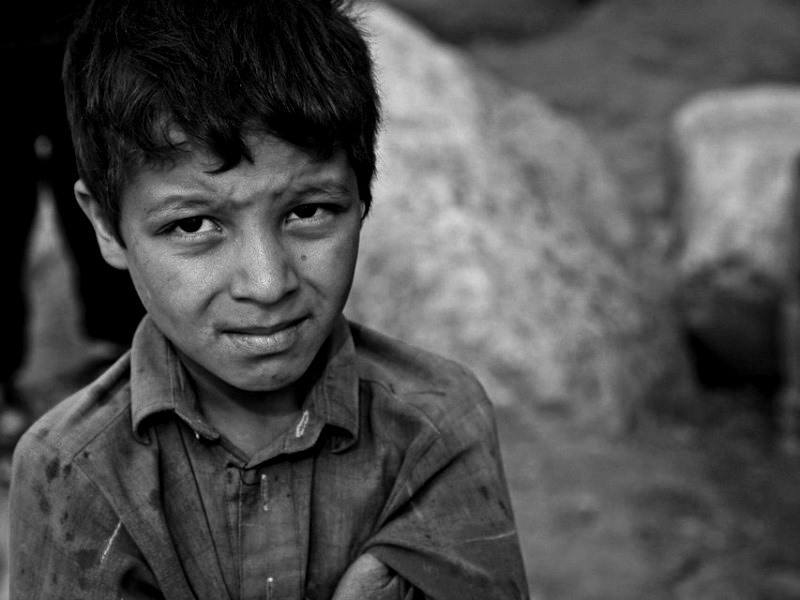


*Micael, 7 years*

Do you choose to donate to the purpose clean water and help victims like Micael?

- Yes
- No [save money to use to whatever you want]

*Scenario 3 – Trafficking, identified*

You will in this scenario decide if you want to donate to an organization that works to decrease trafficking and help victims of trafficking.

You are reading about Monika, 24 years old, a victim of trafficking. She describes that she got tricked to a place when she was at a party, where she was captured by a gang that exploit young women. For 2 years she was sexually abused in several different ways without being able to escape.

You are, while reading about Monikas story, given the possibility to donate to a charity organization that works to minimize cases of trafficking and help victims of trafficking to escape their situation By donating 75 SEK you contribute to educating field workers that actively will work to help victims of trafficking like Monika.


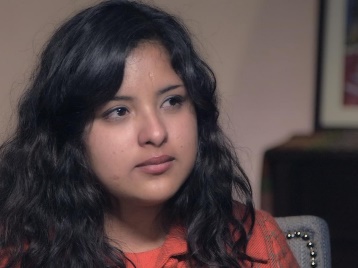
  
*Monika, 24 years*

Do you choose to donate to the purpose trafficking and help victims like Monika?

- Yes
- No [save money to use to whatever you want]

*Scenario 4 – War refugee, identified*

You will in this scenario decide if you want to donate to an organization that helps people who has escaped war.

You are reading about Simon, 34 years old, that recently arrived at a refugee camp together with his kids. Simon fled his country three weeks ago because of a civil war there. When he saw that their lives were in danger, he decided that they needed to escape as soon as possible. He packed fast and they succeeded to get into a neighboring country, where they just recently got access to this refugee camp.

You are reading about a charity organization that works to help children, adults and families who have fled from their home country because of war. They aid these people with shelter, necessities and safety in different refugee camps. By donating 120 SEK you contribute that more refugees like Simon and his children to survive and be helped with accommodation and necessities.


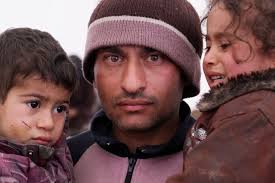


*Simon, 34 years*

Do you choose to donate to the purpose war refugee and help victims like Simon?

- Yes
- No [save money to use to whatever you want]

*Scenario 5 – Diabetes, identified*

You will in this scenario decide if you want to donate to an organization that helps children and adults with diabetes.

You are reading about Niklas, 20 years old, that has had diabetes for several years. He daily needs to think about controlling that his blood sugar won’t get too high or low. Otherwise he risks fainting or to get sick. He goes regularly to doctor appointments to see how his diabetes is and adjust the medication. Niklas feel that his diabetes affects his everyday life much more than his friends that lives without diabetes.

Many children and adults with diabetes are affected negatively in many ways in their everyday life. An organization that works for people with diabetes helps them to get support in their everyday life, for example by facilitate health care visits, create networks and inform about how to ease their everyday life, alleviate their symptoms and prevent serious injuries. This charity organization also contributes to research about diabetes. By donating 85 SEK you contribute to help children and adults like Niklas with diabetes to get support, help and information as well as to research about diabetes.


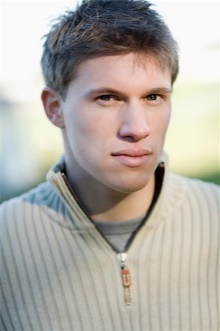


*Niklas, 20 years*

Do you choose to donate to the purpose diabetes and help victims like Niklas?

- Yes
- No [save money to use to whatever you want]

*Scenario 6 – Bullying, identified*

You will in this scenario decide if you want to donate to an organization that works to reduce bullying at schools.

You are reading about Lina, 12 years old, that for 4 years has been bullied at school. It started when she started a new class in lower elementary school. She was quickly picked out and ostracized. Lina says that she also has heard taunts every week from classmates. She always has stomachache when she will go to school and often feels tense during the school day.

You are, while reading about Linas story, about an organization that in different ways works with anti-bullying at schools around the country. This charity organization works with educating and implementing work methods that reduces bullying among children. They are also working to eliminate bullying in specific cases at schools. By donating 100 SEK you contribute to the work with reducing cases of bullying at schools and help victims of bullying like Lina to have a better school situation.


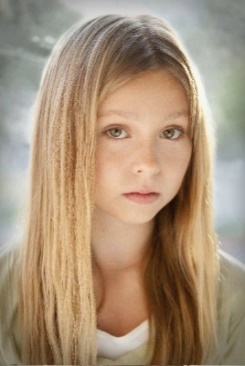


Lina, 12 years

Do you choose to donate to the purpose bullying and help victims like Lina?

- Yes
- No [save money to use to whatever you want]

Conditions descriptive

*Condition 1(no-id, no-oc):* 196 subjects.
50,5% women, 48,0 % men, 1,5% other/do not want to disclose.

*Condition 2 (no-id, oc-reminder:prosocial)* : 188 subjects.
52,1% women, 47,9 % men, 0% other.

*Condition 3 (no-id, oc-reminder:proself):* 192 subjects.
52,6% women, 47,4 % men, 0% other.

*Condition 4 (id, no-oc):* 190 subjects.
51,6% women, 47,9 % men, 0,5% other.

*Condition 5 (id, oc-reminder:prosocial):* 193 subjects.
50,3% women, 48,2 % men, 1,6% other.

*Condition 6 (id, oc-reminder:proself)*: 184 subjects.
47,3% women, 52,2 % men, 0,5% other.

Mean values for demographics (across conditions)

Age: M=45,8, SD=14,5

Educational level: M=3,37, SD=1,43 (range 1:elementary school-5:university 3 year or more)

Political orientation: M=5,10, SD=1,85 (range 1:very far to the left-9:very far to the right)

Household income category: M=2,89, SD=1,46 (range1:0-20k-8:>140k)

Gender: 48,6% (555 st) men, 50, 7% (580 st), 0,7% (8 st) other/don’t want to disclose.

- Excluding participants choosing ”other/don’t want to disclose”: 48,9 % men & 51,1% women.

**Results in figures**

Figure 1 shows the result for the opportunity cost effect on the aggregated level, both for the overall effect across scenarios and separate for single scenarios.

*Figure 1.* Percentage of willingness to donate for opportunity cost reminder, both for the main effect and for single scenarios, across all six decisions.

Figure 2 shows the result for the identifiable victim effect on the aggregated level, both for the overall effect and separate for single scenarios.

*Figure 2.* Percentage of willingness to donate for the identifiable victim effect, both for the main effect and for single scenarios, across all six decisions.

Figure 3 shows that identifying information increased willingness to donate for child victims but not adult victims.

*Figure 3.* Percentage of willingness to donate for the factors identifiability (Non-identified or Identified) and child-adult victimization (Child or Adult). Left figure shows the results for the first decision and right figure for all decisions aggregated.


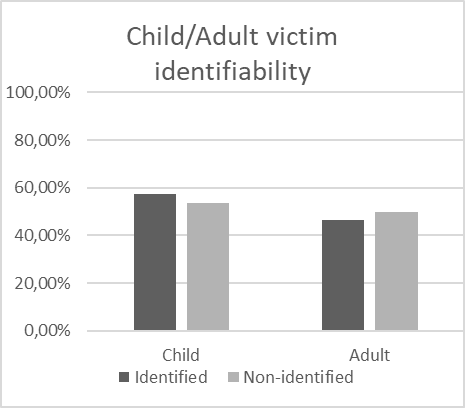

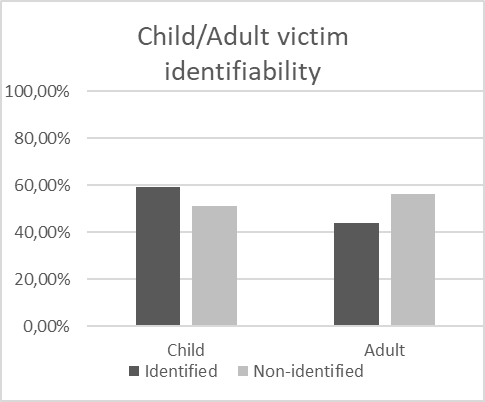


**Rating measures**

These four measures were presented after all six donation decision had been made. Participants then read [inserted relevant description of donation scenario] “In the scenario about [one of the scenarios, e.g. diabetes] you read this [the description from the scenario was visible again for participants, e.g. “You are reading about an organization that…”]”. Then participants were asked to respond to these four questions.

1. When you read this, how positive did you feel?

*A little positive* 1 2 3 4 5 6 *Very positive*

When you read this, how negative did you feel?

*A little negative* 1 2 3 4 5 6 *Very negative*

1. When you read this, how much did it make you think before you made the decision?

*Very little* 1 2 3 4 5 6 *Very much*

1. It is morally right to help this cause.

*Don’t agree at all* 1 2 3 4 5 6 *Agree completely*

1. When you read this, how hard was it to make the choice?

*Very easy* 1 2 3 4 5 6 *Very hard*

We called these measurements for 1) Valence, 2) Think hard, 3) Moral (statement), 4) Decision hardship. The two questions for valence were made into one general valence scale by subtracting negative from positive.

| Below are means, N and standard deviations for the four measurements for each condition. | | | | | |
| --- | --- | --- | --- | --- | --- |
| Conditions | | *Valence* | *Think hard* | *Moral* | *Decision hardship* |
| 1 | Mean | 1,1403 | 2,7500 | 4,3724 | 2,4575 |
|  | N | 196 | 196 | 196 | 196 |
|  | SD | 1,81793 | ,89864 | 1,18665 | ,90875 |
| 2 | Mean | 1,0434 | 2,6152 | 4,4326 | 2,3546 |
|  | N | 188 | 188 | 188 | 188 |
|  | SD | 1,84783 | ,91807 | 1,11059 | ,85584 |
| 3 | Mean | 1,0651 | 2,6276 | 4,5052 | 2,2248 |
|  | N | 192 | 192 | 192 | 192 |
|  | SD | 1,78187 | ,92368 | 1,09748 | ,80607 |
| 4,00 | Mean | -,4114 | 2,5763 | 4,1781 | 2,3544 |
|  | N | 190 | 190 | 190 | 190 |
|  | SD | 1,85137 | ,96539 | 1,25334 | 1,01640 |
| 5,00 | Mean | -,4991 | 2,5734 | 4,3722 | 2,2781 |
|  | N | 193 | 193 | 193 | 193 |
|  | SD | 2,01409 | ,95315 | 1,23524 | ,91483 |
| 6,00 | Mean | -,3288 | 2,5797 | 4,5045 | 2,3125 |
|  | N | 184 | 184 | 184 | 184 |
|  | SD | 1,94447 | 1,04846 | 1,17689 | 1,04818 |
| Total | Mean | ,3405 | 2,6212 | 4,3936 | 2,3307 |
|  | N | 1143 | 1143 | 1143 | 1143 |
|  | SD | 2,01852 | ,95163 | 1,18090 | ,92882 |

*1= no-id, no-oc, 2= no-id, prosocial oc-reminder, 3= no-id, proself oc-reminder, 4=id,
=Number, SD=Standard deviation*

*Conditions: 1= no-id, no-oc, 2= no-id, prosocial oc-reminder, 3= no-id, proself oc-reminder, 4=id, no oc, 5=id, prosocial oc-reminder, 6=id, proself oc-reminder
N=Number, SD=Standard deviation*

Below is the correlation matrix for these measures for each scenario and for the dependent variable.

|  | Valence | Think hard | Moral | Decision hardship |
| --- | --- | --- | --- | --- |
| Breast cancer | .207** | -.047 | .238** | -.072** |
| Clean water | .185** | .024 | .346** | -.001 |
| Trafficking | .171** | -.045 | .352** | -.109** |
| War refugee | .180** | -.025 | .352** | -.065* |
| Diabetes | .219** | -.062* | .232** | -.115** |
| Bullying | .140** | -.047 | .308** | -.124** |
|  |  |  |  |  |
| Valence |  | .025 | .219** | -.039 |
| Think hard |  |  | -.020 | .741** |
| Moral |  |  |  | -.106** |
|  |  |  |  |  |
| Sum of Yes | .277** | -.056 | .458** | -.130** |

***Spearman’s rho.***
**Correlation is significant at 0,01 level (2 tailed).
* Correlation is significant at 0,05 level (2 tailed).

**Results for the question derived from Frederick et al. (2009) about cell phone purchase**

The extra question was given right after the donation decisions and before the rating measures. Participants read

*“Imagine that you have saved some extra money for enjoyments and spontaneous purchases. The electronic store in your local mall has an offer on a cell phone (smart phone). You have wanted to replace your current cell phone for a longer period and now the price is reduced for a cell phone that you are interested in. The cell phone costs* ***4200 SEK.***

***Do you choose to buy the cell phone?***

- *Yes*
- *No [save money to other purchases]”*

|  | |  | | | |
| --- | --- | --- | --- | --- | --- |
|  |  | Oc-reminder | | No oc-reminder | |
|  |  | N | Column N % | N | Column N % |
|  | Yes | 186 | 24,3% | 95 | 25,3% |
|  | No [save money to other purchases] | 581 | 75,7% | 281 | 74,7% |

| **Pearson Chi-Square Tests** | | |
| --- | --- | --- |
|  | | Oc |
|  | Chi-square | ,140 |
|  | df | 1 |
|  | Sig. | ,708 |
|  | | |

In contrast to the results from study 1, where we found a decreased willingness to donate when being reminded of opportunity cost, we found no significant decrease in willingness to purchase when people were reminded of opportunity cost in the question of purchasing a cell phone, *ꭓ^2^* (1) = 0.14, *p* = .71. A likely explanation to the non-significant result is that opportunity cost had become salient for all participants by the time they answered this question, since they had answered the six donation decisions right before the received this question. Thus, the effect of being reminded of opportunity cost did not make a significant effect at this time.

**Additional tests for study 1 for identifiability and frames**

We tested for a possible interaction effect between opportunity cost frame (stated as spending on other charities or stated as spending on anything else) and identifiability (identified versus non-identified). No significant results emerged from these analyses, both for the first decision, *ꭓ^2^* (3) = 1.20, *p* = .75, and for the main effect on aggregated level, *ꭓ^2^* (3) = 0.88, *p* = .83.

**Study 2**

*Scenario – unidentified, control/prosocial frame/proself frame*

Imagine that you are at the check-out in a store and are being asked to donate to a charity organization that works to reduce bullying in schools. The organization works with anti-bullying at schools around the country by educating and implementing work methods that reduces bullying among children. They are also working to eliminate bullying in specific cases at schools. By donating, you contribute to the work with reducing cases of bullying at schools and help victims of bullying to have a better school situation.

Do you choose to donate?

- Yes, I want to donate
- No, I don’t want to donate/ No, I’ll save the money to spend on other charitable causes / No, I’ll save the money to spend on my future purchases

*Scenario – identified, control/prosocial frame/proself frame*

Imagine that you are at the check-out in a store and are being asked to donate to a charity organization that works to reduce bullying in schools. The charity organization describes Lina, 12 year, that for 4 years has been bullied at school. It started when she started a new class in lower elementary school. She was quickly picked out and ostracized. Lina says that she also has heard taunts every week from classmates. She always has stomachache when she will go to school and often feels tense during the school day. The charity organization works with anti-bullying at schools around the country by educating and implementing work methods that reduces bullying among children. They are also working to eliminate bullying in specific cases at schools. By donating, you contribute to the work with reducing cases of bullying at schools and help victims of bullying like Lina to have a better school situation.

(Lina, 12 years)


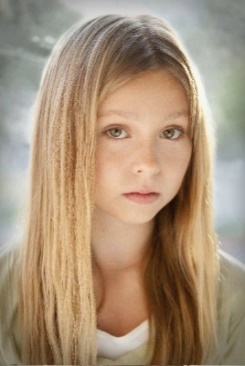


Do you choose to donate and help victims like Lina?

- Yes, I want to donate
- No, I don’t want to donate/ No, I’ll save the money to spend on other charitable causes / No, I’ll save the money to spend on my future purchases

**Additional tests for study 2 on interaction between framing and identifiability**

We tested for a possible interaction effect between opportunity cost frame (stated as spending on other charities or stated as spending on anything else) and identifiability (identified versus non-identified). The result shows a significant effect, *ꭓ^2^*(3) = 19.5, *p* < .001. The result show that a proself framed reminder, compared to a prosocial framed reminder, decreased willingness to donate for non-identified victims (from 50.5% to 49.0%) whereas it increased it for identified victims (from 63.0% to 66.3%).

There was no significant interaction effect in donated amount between the frame of the opportunity cost reminder and identifiability, *F*(3, 474) = 0.54, *p* = .66. However, when including non-donors, the result was significant, *F*(3, 832) = 3.39, *p* = .018, *η_p_^2^* = .012. Bonferroni post-hoc test shows that participants who received a reminder that was framed prosocial and who did not see an identified victim (*M* = 43.6, *SD* = 68.8) donated significantly less than participants who received a proself framed reminder and saw an identified victim (*M* = 67.7, *SD* = 96.4).
